# Supplementary material for: The Metabolic Potential of Endophytic Actinobacteria Associated with Medicinal Plant Thymus roseus as a Plant-Growth Stimulator
Source: Microorganisms. 2022 Sep 7;10(9):1802. doi: 10.3390/microorganisms10091802 (PMC9505248; doi:10.3390/microorganisms10091802)
Supplement: Supplementary file 1 [file microorganisms-10-01802-s001.zip › Tables S1-S6.pdf]

Table S1: The beneficial traits in selected endophytic bacteria from *Thymus roseus in vitro*.

Strain Code	Species	Exo-enzymes			Nitrogen Fixation Capacity[d]		Phosphorus[e]	Siderophore production[f]	IAA[g]	Chitinase
		Protase[a]	Cellulase[b]	Lipase[c]	NFB	ASHBY				
**XIEG05**	*Saccharopolyspora gregorii*	2.194	2.260	+++	+	+	+	+	0.675	+++
**XIEG07**	*Streptomyces enissocaesilis*	++	++++	++	+	+	+	-	0.390	+++
**XIEG10**	*Streptomyces gulbargensis*	2.810	2.889	+++	+	+	+	+	1.133	+++
**XIEG34**	*Streptomyces thermoviolaceus`*	+	++	-	+	+	+	1.81212	0.649	++
**XIEG40**	*Streptomyces drozdowiczii*	3.470	3.636	+++	+	+	+	+	0.313	-
**XIEG41**	*Streptomyces olivaceus*	+	2.744	+++	+	+	++	+	0.279	+
**XIEG45**	*Streptomyces enissocaesilis*	++	2.812	+++	+	+	+	+	0.700	+++
**XIEG50**	*Streptomyces luteus*	++	3.044	+++	+	+	++	+	0.644	++
**XIEG55**	*Streptomyces viridochromogenes*	++	2.200	++	+	+	+	+	0.229	++++
**XIEG63**	*Saccharopolyspora taberi*	1.487	++	++	+	+	+	+	0.262	-

[a]Protease production: "-"no production; "+" weak halo around the colony; "++" clear halo around the colony (1.00-2.00]; "+++" that strong halo around the colony (2.00-3.00]. [b]Cellulose production: "-"no production; "+" weak halo around the colony (1.00-2.00]; "++" clear halo around the colony (2.00-4.00]; "+++" that strong halo around the colony (4.00-6.00]. [c]Lipase production: "-"no production; "+" weak halo around the colony (1.00); "++" clear halo around the colony (1.00-3.00); "+++" that strong halo around the colony (3.00-5.00). [d]Nitrogen-fixing, "+" indicate the growth. [e]Phosphorus: "-"no production; "+" weak halo around the colony (1.00-1.50); "++" clear halo around the colony (1.50-2.00); [f]Siderophore: "-"no ability; "+" indicates that bacteria can grow on this medium; "++" clear halo around the colony (1.00-2.00]. [g]IAA = production of the plant hormone indole-3-acetic acid. Pink color indicated indole production. Values in parentheses refer to the absorbance at 530 nm: CK value of OD530 of 0.08 indicates no production; results (0.08-2.5).

Table S2: The beneficial traits in selected endophytic bacteria from *Thymus roseus in vitro*.

Strain Code	Species	Inhibition rate/% (Inhibition activity)	
		Tomato	Cotton
**XIEG05**	*Saccharopolyspora gregorii*	45.43	20.83
**XIEG07**	*Streptomyces enissocaesilis*	61.57	17.98
**XIEG10**	*Streptomyces gulbargensis*	+	+
**XIEG41**	*Streptomyces olivaceus*	33.80	15.18
**XIEG43**	*Streptomyces setonii*	+	12.5
**XIEG44**	*Nocardiopsis dassonvillei*	41.03	16.83
**XIEG45**	*Streptomyces enissocaesilis*	65.66	41.38
**XIEG47**	*Streptomyces pratensis*	41.57	+
**XIEG50**	*Streptomyces luteus*	39.47	++
**XIEG51**	*Streptomyces luteus*	59.40	44.83
**XIEG61**	*Nocardiopsis alba*	42.39	11.68
**XIEG62**	*Nocardiopsis alba*	41.59	13.96

Table S3: GC-MS identified components of the antibiosis crude extract of XIEG05 and *V. dahliae* mixture at pH7. Volatile Compounds are listed in ascending order of Retention with at least percentage match ≤70%.

NO	Retention Time (min)	Compounds	Percentage Match %	Molecular formula	Molecular Weight
1	3.387	o-Xylene	95	C_8H_{10}	106.078
2	3.753	Styrene	95	C_8H_8	104.15
3	8.134	Phenylethyl Alcohol	94	C_8H_{100}	122.073
4	13.414	trans-1,10-Dimethyl-trans-9-decalol	96	$C_{12}H_{22}O$	182.167
5	21.192	Dibutyl phthalate	95	$C_{16}H_{22}O_4$	278.152
6	25.939	Cyclotetracosane	95	$C_{24}H_{48}$	336.376
7	26.222	Phenol, 2,2'-methylenebis[6-(1,1-dimethylethyl)-4-methyl-	95	$C_{69}H_{93}O_6P$	340.24
8	27.888	9-Tricosene, (Z)-	94	$C_{23}H_{46}$	322.36
10	27.988	Oleyl alcohol, trifluoroacetate	95	$C_{20}H_{35}F_3O_2$	364.259
11	29.704	Pyridine-3-carboxamide, oxime, N-(2-trifluoromethylphenyl)-	90	$C_{13}H_{10}F_3N_3O$	281.078
12	30.636	Decahydro-8a-ethyl-1,1,4a,6-tetramethylnaphthalene	72	$C_{16}H_{30}$	222.235
13	32.519	Octasiloxane, 1,1,3,3,5,5,7,7,9,9,11,11,13,13,15,15-hexadecamethyl-	91	$C_{16}H_{48}O_7Si_8$	578.171

Table S4: GC-MS identified components of the crude extract of XIEG07 at pH7. Volatile compounds are listed in ascending order of Retention Time with at least percentage match ≤70%).

NO	Retention Time (min)	Compounds	Percentage Match %	Molecular formula	Molecular Weight
1	3.254	Ethylbenzene	91	C_8H_{10}	106.078
2	3.37	p-Xylene	97	C_8H_{10}	106.078
3	8.117	Phenylethyl Alcohol	94	C_8H_{100}	122.073
4	8.217	Methyl pyrrole-2-carboxylate	87	$C_6H_7NO_2$	125.048
6	9.816	Dodecane	96	$C_{12}H_{26}$	170.203
7	13.397	trans-1,10-Dimethyl-trans-9-decalol	97	$C_{12}H_{22}O$	182.167
8	18.394	Pyrrolo[1,2-a]pyrazine-1,4-dione, hexahydro-	95	$C_{10}H_{16}N_2O_2$	154.074
9	19.477	N-Acetyltyramine	76	$C_{10}H_{13}NO_2$	179.095
10	21.192	Dibutyl phthalate	95	$C_{16}H_{22}O_4$	278.152
11	27.872	Oleyl alcohol, trifluoroacetate	95	$C_{20}H_{35}F_3O_2$	364.259
12	27.988	Oleyl alcohol, trifluoroacetate	95	C_8H_9NO	364.259
13	29.704	2,6,10,14-Tetramethyl-7-(3-methylpent-4-enylidene) pentadecane	80	$C_{25}H_{48}$	348.376
14	31.969	Octasiloxane, 1,1,3,3,5,5,7,7,9,9,11,11,13,13,15,15-hexadecamethyl-	87	$C_{16}H_{48}O_7Si_8$	578.171
15	32.819	Heptasiloxane, 1,1,3,3,5,5,7,7,9,9,11,11,13,13-tetradecamethyl-	74	$C_{14}H_{42}O_6Si_3$	504.152
16	33.568	Octasiloxane, 1,1,3,3,5,5,7,7,9,9,11,11,13,13,15,15-hexadecamethyl-	91	$C_{16}H_{48}O_7Si_8$	578.171

Table S5: GC-MS identified components of the antibiosis crude extract of XIEG45 and *V. dahliae* mixture at pH7. Volatile Compounds are listed in ascending order of Retention Time with at least percentage match ≤70%.

NO	Retention Time (min)	Compounds	Percentage Match %	Molecular formula	Molecular Weight
1	4.719	Pentanoic acid, 4-methyl-	90	$C_6H_{12}O_2$	116.084
2	7.651	Phenol, 2-methoxy-	94	$C_{10}H_{12}O_2$	124.052
3	8.067	Maltol	93	$C_6H_6O_3$	126.032
4	8.15	Phenylethyl Alcohol	93	C_8H_{100}	122.073
5	8.683	4H-Pyran-4-one, 2,3-dihydro-3,5-dihydroxy-6-methyl-	95	$C_6H_8O_4$	144.042
6	10.732	Benzeneacetic acid	95	$C_8H_8O_2$	136.052
7	13.747	Phenol, 3,4-dimethoxy-	95	$C_8H_{10}O_3$	154.063
8	20.726	Pyrrolo[1,2-a]pyrazine-1,4-dione, hexahydro-3-(2-methylpropyl)-	94	$C_{10}H_{16}N_2O_2$	210.137
9	21.192	Dibutyl phthalate	94	$C_{16}H_{22}O_4$	278.152
10	27.888	13-Tetradecen-1-ol acetate	87	$C_{16}H_{30}O_2$	254.225
11	29.704	Octasiloxane, 1,1,3,3,5,5,7,7,9,9,11,11,13,13,15,15-hexadecamethyl-	76	$C_{16}H_{48}O_7Si_8$	578.171
12	31.969	Heptasiloxane, 1,1,3,3,5,5,7,7,9,9,11,11,13,13-tetradecamethyl-	74	$C_{14}H_{42}O_6Si_7$	504.152
13	32.519	Heptasiloxane, 1,1,3,3,5,5,7,7,9,9,11,11,13,13-tetradecamethyl-	99	$C_{14}H_{42}O_6Si_7$	

Table S6: GC-MS identified components of the antibiosis crude extract of XIEG51 and *V. dahliae* mixture at pH7. Volatile Compounds are listed in ascending order of Retention Time with at least percentage match ≤70%.

NO	Retention Time (min)	Compounds	Percentage Match %	Molecular formula	Molecular Weight
1	3.37	Benzene, 1,3-dimethyl-	95	$C_8H_{10}Cl_6$	106.078
2	3.77	o-Xylene	95	C_8H_{10}	106.078
3	8.067	Maltol	93	$C_6H_6O_3$	126.032
4	8.117	Phenylethyl Alcohol	94	C_8H_{100}	122.073
5	8.683	4H-Pyran-4-one, 2,3-dihydro-3,5-dihydroxy-6-methyl-	91	$C_6H_8O_4$	144.042
6	10.732	Benzeneacetic acid	91	$C_8H_8O_2$	136.052
7	13.664	Benzeneethanol, 4-hydroxy-	87	$C_{14}H_{13}N_3O_2$	138.068
8	13.764	Phenol, 3,4-dimethoxy-	95	$C_8H_{10}O_3$	154.063
10	18.577	Tryptophol	95	$C_{10}H_{11}F_3O_2$	161.084
11	20.709	Pyrrolo[1,2-a]pyrazine-1,4-dione, hexahydro-3-(2-methylpropyl)-	95	$C_{13}H_{10}F_3NO$	210.137
12	21.192	Dibutyl phthalate	72	$C_{16}H_{22}O_4$	278.152
13	23.907	Eicosane	95	$C_{20}H_{42}$	282.329
14	24.957	Heneicosane	97	$C_{21}H_{44}$	296.344
15	25.989	Tetracosane	97	$C_{24}H_{50}$	338.391
16	26.223	Phenol, 2,2'-methylenebis[6-(1,1-dimethylethyl)-4-methyl-	95	$C_{69}H_{93}O_6P$	340.24
17	27.888	Nonadecyl trifluoroacetate	94	$C_{21}H_{39}F_3O_2$	380.29
18	27.972	1-Docosene	95	$C_{22}H_{44}$	308.344
19	31.419	Octasiloxane, 1,1,3,3,5,5,7,7,9,9,11,11,13,13,15,15-hexadecamethyl-	87	$C_{16}H_{48}O_7Si_8$	578.171
