# Supplementary figures and images for: The Metabolic Potential of Endophytic Actinobacteria Associated with Medicinal Plant Thymus roseus as a Plant-Growth Stimulator
Source: Microorganisms. 2022 Sep 7;10(9):1802. doi: 10.3390/microorganisms10091802 (PMC9505248; doi:10.3390/microorganisms10091802)

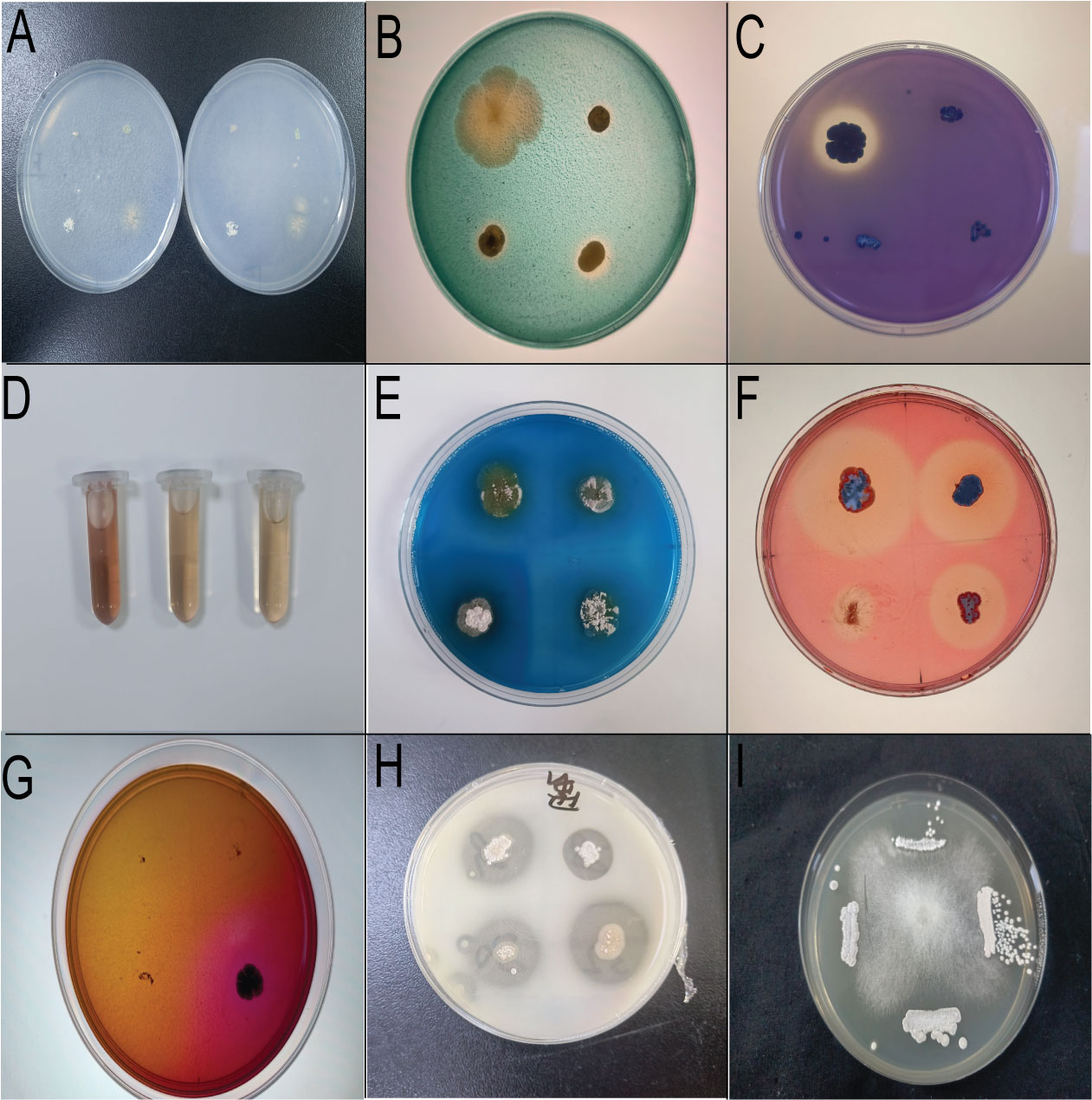

Supplement: Supplementary file 1 [file microorganisms-10-01802-s001.zip › Figure S1 PGP photos.jpg]

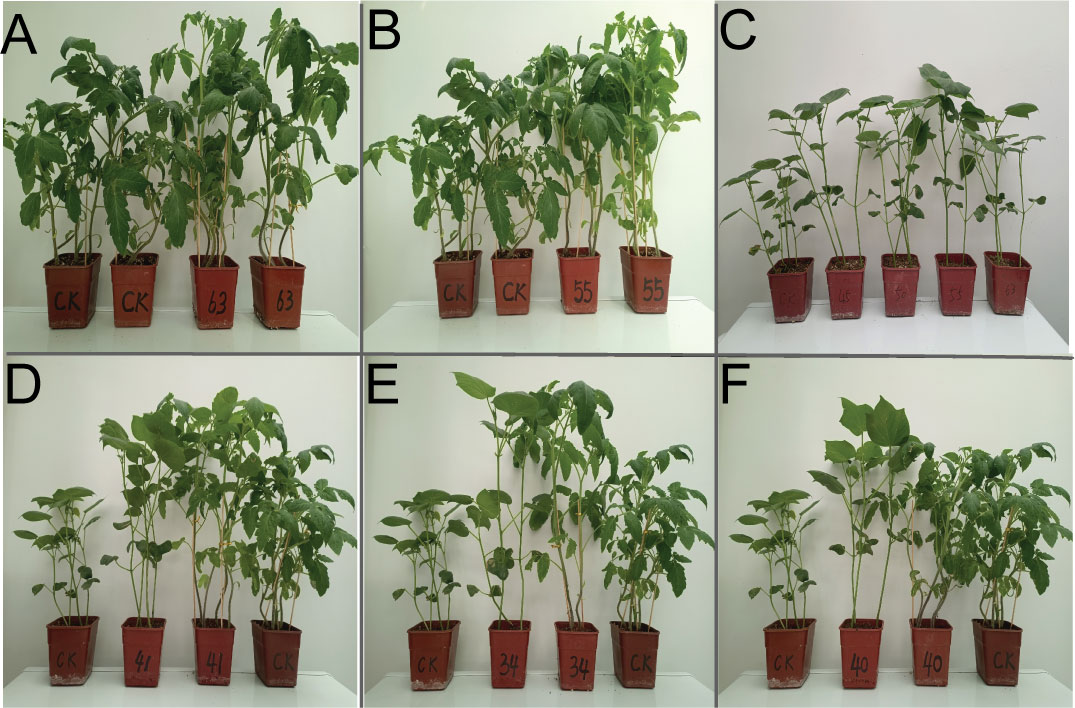

Supplement: Supplementary file 1 [file microorganisms-10-01802-s001.zip › Figure S2 plant photos.jpg]
